# Case Report: Complete Remission With Anti−PD−1 and Anti−VEGF Combined Therapy of a Patient With Metastatic Primary Splenic Angiosarcoma

**DOI:** 10.3389/fonc.2022.809068

**Published:** 2022-03-03

**Authors:** Weiran Xu, Kai Wang, Wenguang Gu, Xinxin Nie, Hao Zhang, Chuanhao Tang, Li Lin, Jun Liang

**Affiliations:** ^1^ Department of Oncology, Peking University International Hospital, Beijing, China; ^2^ Department of Laboratory Medicine, Beijing Haidian Hospital, Beijing, China; ^3^ Department of Medicine, Geneplus-Beijing, Beijing, China; ^4^ Department of Medical Affairs, Shanghai Junshi Biosciences Co., Ltd., Beijing, China

**Keywords:** primary splenic angiosarcoma, PD-1 inhibitor, immunotherapy, anti-VEGF therapy, complete remission

## Abstract

Primary splenic angiosarcoma (PSA) is a rare malignancy with poor prognosis. At present, little study is available on immunotherapy in PSA. Here, we report a case of a patient with metastatic PSA who was treated with programmed death-1 (PD-1) inhibitors and vascular endothelial growth factor (VEGF) tyrosine kinase inhibitors combined therapy and achieved complete response (CR). The patient was a 57−year−old woman with three liver metastases. She was treated with seven cycles of toripalimab plus anlotinib. Programmed death-ligand 1 (PD-L1) immunohistochemistry and next-generation sequencing was performed, and the PD-L1 tumor proportion score was 75%. Finally, she achieved CR after six cycles of the combined therapy regimen. No serious adverse events were detected. To the best of our knowledge, this is the first clinical evidence that anti-PD-1 plus anti-VEGF therapy might be a promising option for patients with metastatic PSA. However, more clinical trials are needed to verify this conclusion.

## Introduction

Primary splenic angiosarcoma (PSA) is a rare and aggressive tumor with poor prognosis and a high rate of liver metastasis. The typical symptom of PSA is left upper abdomen pain (1–3); other symptoms include weakness or fatigue, fever, chest pain, weight loss, and bleeding (1, 4). Splenectomy is the only potentially curative treatment for patients with early-stage PSA. In addition, some patients with distant metastasis may receive emergency splenectomy due to splenic rupture.

Although some case reports have reported the potential benefit of systemic therapy in PSA, traditional chemotherapy have limited efficacy in metastatic PSA (1, 5). In recent years, programmed death-1 (PD-1) inhibitors have significantly improved the long-term survival of patients with various tumors (6, 7). Moreover, vascular endothelial growth factor (VEGF) tyrosine kinase inhibitors (TKIs) have shown promising effects in patients with angiosarcoma (8).

Here, we report a case of a metastatic PSA patient with high expression of programmed death-ligand 1 (PD-L1) who reached complete response (CR) after anti−PD−1 and anti−VEGF combination therapy.

## Case Report

The patient was a 57−year−old woman who was diagnosed with PSA in 2020. She presented to a local hospital with left-sided upper abdominal pain for three hours.

**Figure 1:**
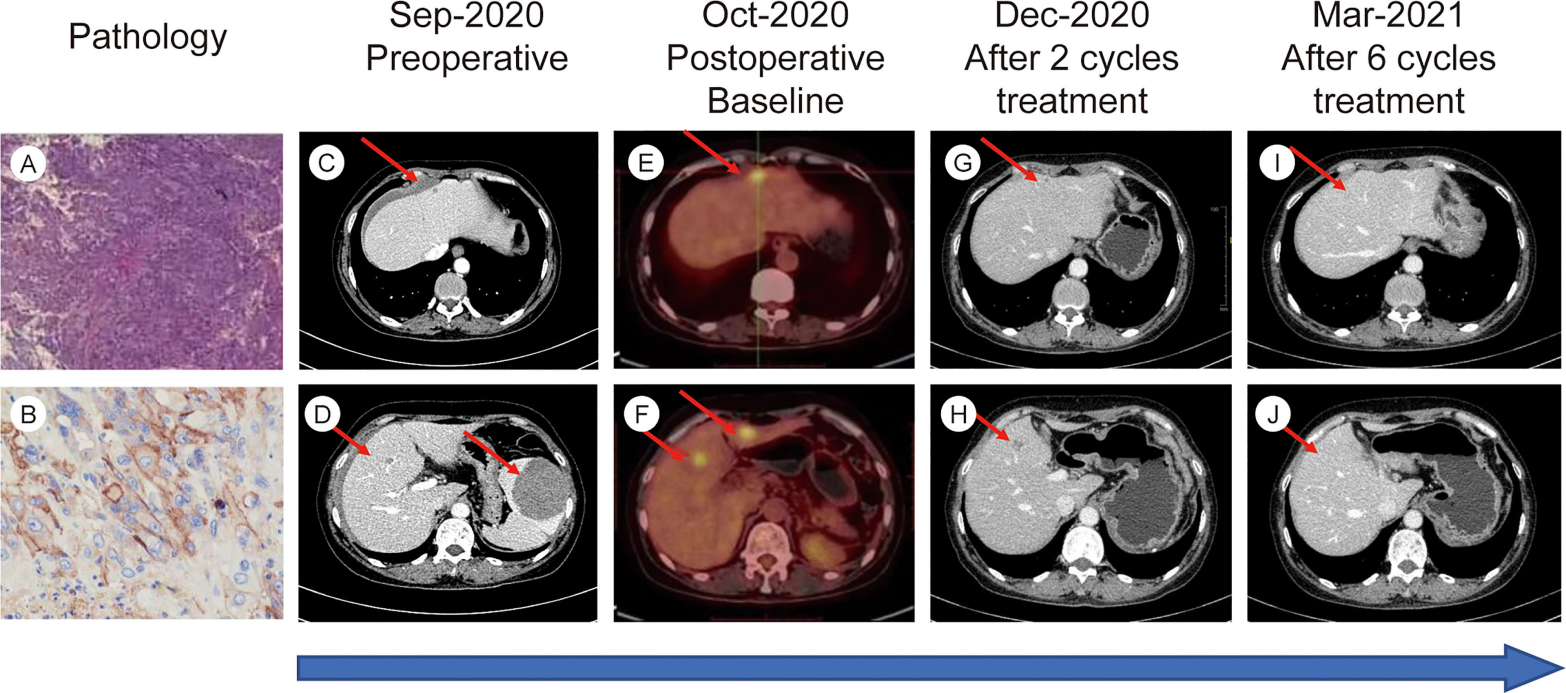
**(A)** H&E stain from postoperative tumor tissue (10X magnification). **(B)** Immunohistochemical staining for PD-L1 in postoperative tumor tissue. **(C–J)** Timelines of imaging changes.

There was no bloating, nausea, or vomiting. No family cancer history was noted. Abdominal contrast-enhanced computed tomography (CT) showed spontaneous rupture of a spleen neoplasm and abdominal hemorrhage, three suspicious lesions in the liver were also detected. One day later, an emergency splenectomy was performed. The postoperative immunohistochemical staining results were as follows: CD31(+), CD34(+), EGFR (+), CK (−), P63(−), CK20(−), CK5/6(−), Syn (−), and CK7(−). The final pathologic results confirmed the diagnosis of angiosarcoma (**Figure 1**). Positron emission tomography-CT (PET-CT) was performed 1 month after surgery, demonstrating three hypermetabolic foci in the liver, which were diagnosed as hepatic metastases and correspond to the same lesions initially found in CT. No other distant metastases were identified.

One month later, she was referred to our hospital for further care. PD-L1 staining with a 22C3 antibody was performed, and the results indicated high PD-L1 expression [tumor proportion score (TPS)=75%]. Moreover, we performed next-generation sequencing of 1021 cancer-related genes using tumor tissue and matched lymphocyte samples. The results suggested microsatellite-stable (MSS). No germline pathogenic or likely pathogenic variants were identified, but eight somatic mutations (MYC, TP53, TSC2, BR*D2, MAP2K4, NCOR1, PTEN, FAS*) were found in this patient. She finally received combined anti-VEGF and anti-PD-1 inhibitor treatment in 3-week cycles, with 240 mg toripalimab admitted intravenously on day 1 of each cycle and 10 mg anlotinib given daily on days 1 to 14 of each cycle. Adverse reactions, including grade 1 myelosuppression and grade 2 diarrhea, were noted. However, no serious adverse events were observed in this patient.

The efficacy of the combination treatment was assessed using CT and magnetic resonance imaging scans. Tumor load was evaluated at baseline and after every two cycles of treatment using response evaluation criteria in solid tumors (RECIST) guidelines (version1.1). In total, seven cycles of the combined treatment were delivered. The patient exhibited a favorable response to our combined regimen. The evaluation of efficacy after two and four cycles was partial response. The tumor shrank approximately 70% after four cycles of treatment. Surprisingly, our patient finally achieved CR after six cycles of treatment (**Figure 1**). PET-CT was performed to confirm CR after the seventh cycle, and no 18F-fluorodeoxyglucose elevation was detected in the original metastatic sites. The patient declined further treatment for personal reasons. Three months after the final cycle of treatment, imaging examinations revealed no evidence of tumor relapse. As the influence of COVID-19, the patient did not come to our hospital regularly for re-examination since then.

## Discussion

PSA is a kind of malignancy that derived from the splenic vascular endothelium with an extremely low incidence. Fewer than 300 cases of PSA have been reported to date. The average age at diagnosis is 50–60 years (9, 10). As previously reported, the median survival time of metastatic PSA is approximately one year. Rupture of the spleen and distant metastasis are considered poor prognostic factors (11).

Because of the rarity of the disease, there is no standard treatment protocol. Furthermore, there are no randomized clinical trial data to support a systemic treatment regimen for metastatic PSA. Based on the literature of other sarcomas, some researchers have attempted several first-line chemotherapy regimens, including paclitaxel, anthracycline, doxorubicin, and ifosfamide (12–15). Unfortunately, the overall response rate was relatively low.

Combination treatment with antiangiogenic drugs and immune checkpoint inhibitors has been demonstrated to be effective in multiple malignancies (16). The underlying mechanism involves normalization of the tumor vessels through anti-VEGF therapy, which might improve the infiltration of tumors by activating effector T cells and subsequently convert the immunosuppressive tumor microenvironment (TME) into an immune-active TME (17, 18).

There have been several case reports regarding the combination therapy of antiangiogenic drugs and immune checkpoint inhibitors in sarcomas. A patient with metastatic undifferentiated pleomorphic sarcoma received pembrolizumab and pazopanib after multiple lines of therapy and had a partial response for 9 months (19). In addition, a patient with recurrent intestinal follicular dendritic cell sarcoma received sintilimab plus lenvatinib as third-line treatment and achieved a progression-free survival of 7 months (20). Several clinical trials have also explored the combination therapy in sarcoma. A phase II single-arm study of pembrolizumab plus lenvatinib in previously treated classic Kaposi sarcoma is in progress (https://www.clinicaltrialsregister.eu/ctr-search/search?query=2020-004426-36).Toripalimab is a newly developed monoclonal antibody that blocks PD-1. Clinical trial data have exhibited a promising antitumor activity of toripalimab in metastatic sarcoma and other malignancies (21, 22). Anlotinib is a novel TKI targeting VEGF1-3 and has shown encouraging effects in sarcoma (23, 24). A Phase II clinical trial (NCT04172805) is aimed to test the safety and effectiveness of anlotinib and toripalimab in soft tissue sarcoma. However, the results have not been published (https://clinicaltrials.gov/ct2/show/NCT04172805). In our patient, we innovatively attempted toripalimab and anlotinib combined therapy for metastatic PSA, and the regimen was surprisingly effective and well-tolerated.

High expression of PD-L1 predicts better efficacy of immunotherapy in several cancers (25, 26). A previous study found that the positive rate of PD-L1 was approximately 60% in angiosarcoma, and the differentiation level of the tumor was significantly associated with the PD-L1 status (27). The TPS of our patient was as high as 75% and the tumor rapidly decreased in size after several cycles of combined treatment.

**Figure 2:**
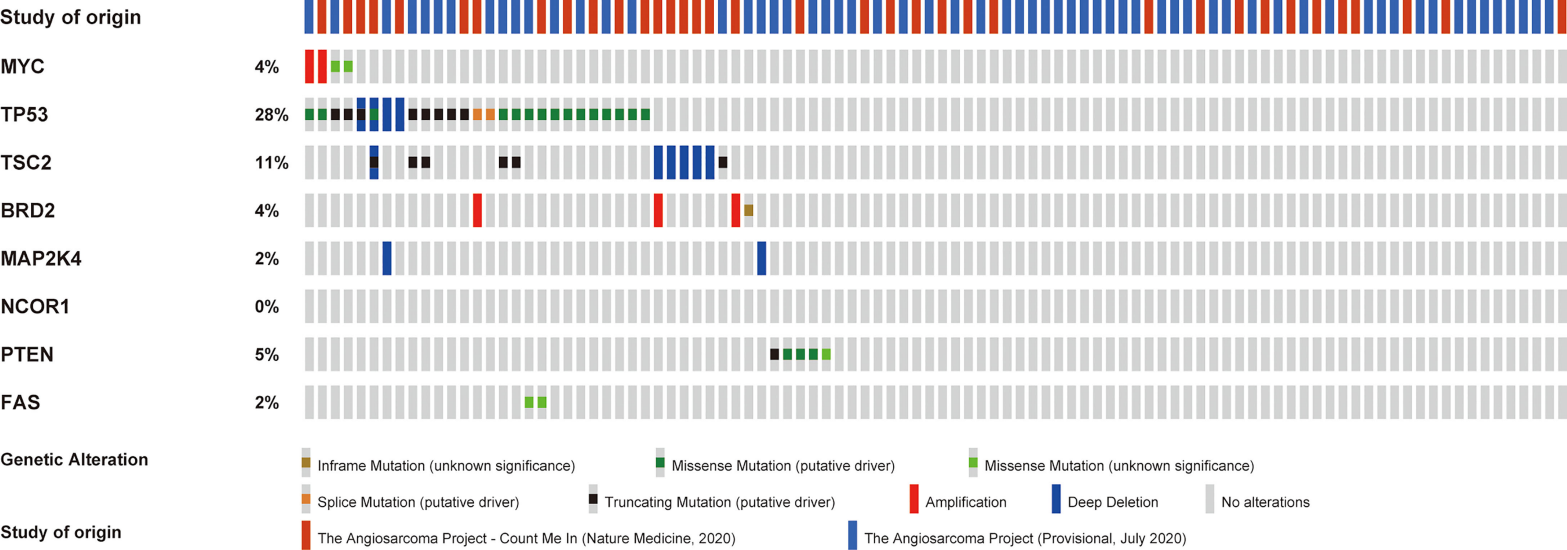
Analysis of the patients’ mutation genes in TCGA sarcoma data.

**Figure 3:**
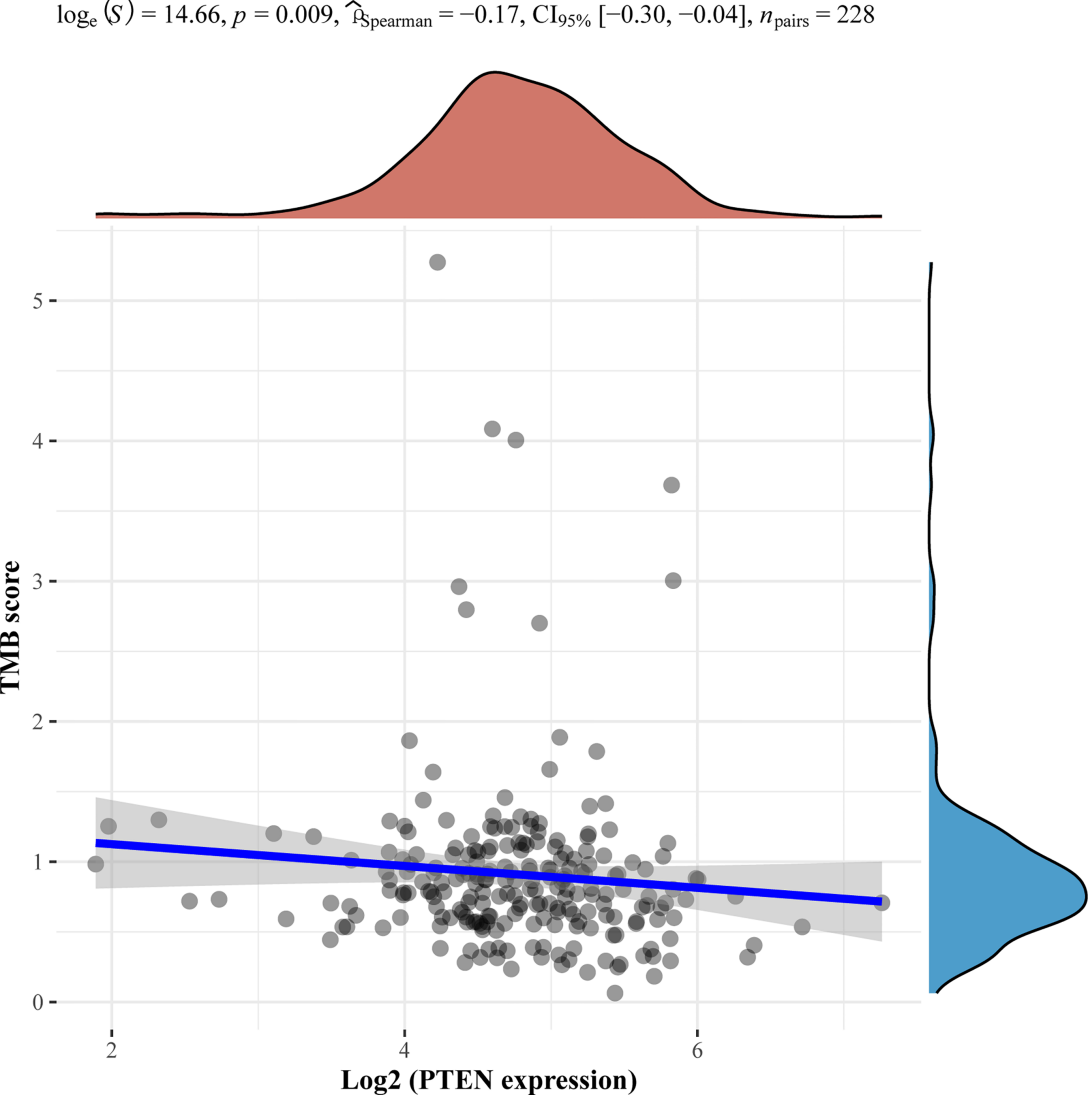
Correlation analysis of PTEN gene expression and TMB. The horizontal axis in the figure represents the expression distribution of the gene, and the ordinate is the expression distribution of the TMB score. The density curve on the right represents the distribution trend of the TMB score; the upper density curve represents the distribution trend of the gene; the top side represents the correlation p value, correlation coefficient and correlation calculation method.

Of note, we performed next-generation sequencing of tumor tissue and further analyzed angiosarcoma datasets from The Cancer Genome Atlas (TCGA). TCGA data showed the mutation frequencies of these genes in angiosarcoma patients (**Figure 2**). We further analyzed the correlation between the expression of these genes and the tumor mutational burden (TMB) in angiosarcoma cases from the TCGA database. Finally, we observed that *PTEN* mutation was negatively correlated with TMB (**Figure 3**).

We also found that some gene mutations of our patients were correlated with the efficacy of immunotherapy. *TSC2* was associated with T cell exhaustion inhibition, which upregulated PD-L1 on tumors (28). The *TP53* mutation status was related to the survival benefit of PD-1 inhibitors in non-small cell lung cancer (29). *NCOR1* mutation was reported as a potential positive biomarker to predict the efficacy of immunotherapy in bladder cancer (30). However, we also identified negative predictors of immunotherapy in our patient, such as *PTEN* mutation (31). This gene alteration reduces CD8-positive T cells in the immune microenvironment, leading to an inadequate response to PD-1 inhibitors.

Surprisingly, our patient quickly achieved CR during toripalimab plus anlotinib treatment and had no serious adverse events. Our case suggests that anti-PD-1 plus anti-VEGF therapy might be a promising option for metastatic PSA patients with high expression of PD-L1. However, clinical trials are warranted to confirm the efficacy of this regimen for these patients.

## Data Availability Statement

The original contributions presented in the study are included in the article/supplementary material. Further inquiries can be directed to the corresponding authors.

## Ethics Statement

Ethical approval was obtained from the Peking University International Hospital Research Ethics Committee. The patient provided her written informed consent to participate in this study. The patients/participants provided their written informed consent to participate in this study. Written informed consent was obtained from the individual(s) for the publication of any potentially identifiable images or data included in this article.

## Author Contributions

WX drafted the manuscript. XN revised the manuscript. All authors read and approved the final manuscript.

## Conflict of Interest

Author WG was employed by Geneplus-Beijing. Co-author XN and HZ were full-time employee of Shanghai Junshi Biosciences Co., Ltd.

The remaining authors declare that the research was conducted in the absence of any commercial or financial relationships that could be construed as a potential conflict of interest.

## Publisher’s Note

All claims expressed in this article are solely those of the authors and do not necessarily represent those of their affiliated organizations, or those of the publisher, the editors and the reviewers. Any product that may be evaluated in this article, or claim that may be made by its manufacturer, is not guaranteed or endorsed by the publisher.
